# Case Report: Innovative anesthetic approaches for whole lung lavage in an infant with pulmonary alveolar proteinosis

**DOI:** 10.3389/fped.2025.1496553

**Published:** 2025-01-29

**Authors:** Jiaxiang Chen, Xiaoli Shi, Youbing Tu, Yuanzhen Chen, Xueqing Wang, Jing Shen, Liang Xu, Ligang Meng

**Affiliations:** Department of Anesthesiology, Shenzhen Children’s Hospital, Shenzhen, China

**Keywords:** whole lung lavage, pulmonary alveolar proteinosis, pediatric anesthesia, bronchial blocker, one-lung ventilation

## Abstract

**Introduction:**

Pulmonary alveolar proteinosis (PAP) is a rare disease in infancy characterized by the accumulation of lipoprotein material within the alveoli, leading to impaired gas exchange, ventilation-perfusion mismatch, and, in severe cases, respiratory failure that may result in death. Treatment options include medical therapy and whole lung lavage (WLL), typically requiring lung isolation techniques or extracorporeal membrane oxygenation. Previous studies have reported the application of several lung isolation techniques in pediatric WLL. However, the use of a bronchial blocker (BB) in infant WLL has not been previously reported.

**Case description:**

This study reports the anesthesia management of a 12-month-old infant diagnosed with secondary PAP, complicated by severe pneumonia and patent ductus arteriosus. The child presented with respiratory failure requiring WLL. The anesthesia method employed was intravenous general anesthesia, and airway management involved using a BB placed outside the endotracheal tube to facilitate one-lung ventilation (OLV). The procedure successfully maintained blood oxygen levels above 90%, and the WLL was completed without any anesthetic complications.

**Conclusion:**

This case demonstrates that using endotracheal intubation combined with extraluminal placement of a BB for lung isolation is a viable and effective approach for performing WLL in infants.

## Introduction

Pulmonary alveolar proteinosis (PAP) is a rare lung disorder characterized by the accumulation of surfactant, a lipoprotein-rich material, within the alveoli—the tiny air sacs in the lungs (1, 2). The progressive accumulation of excess surfactant in the alveolar spaces impairs gas exchange and gradually depletes respiratory reserves. This can lead to respiratory failure, and if left untreated, may result in death (3). The disease is classified into primary, secondary and congenital forms. The current standard of care for both primary PAP and secondary PAP is whole lung lavage (WLL) (4). Effective airway management during WLL is critical and typically involves one-lung ventilation (OLV) techniques, such as double-lumen endotracheal tubes (DLTs), bronchial blocker (BB), or, in some cases, extracorporeal membrane oxygenation for respiratory support. DLTs are most commonly used in WLL, but the smallest size is 26F, which is generally designed for use in children over 6 years old. For the infant population, WLL requires careful selection of lung isolation techniques that are tailored to the child's airway size and anatomy (5). Infants have higher oxygen consumption rates and lower functional residual capacities, making them more susceptible to hypoxemia during OLV (6, 7). We present case reports detailing novel techniques used for an infant undergoing WLL at our institution, offering new insights into anesthesia management for this procedure.

## Case description

A 12-month-old female infant, weighing 7 kg, was admitted to the hospital with unexplained rapid breathing persisting for more than two weeks. Upon admission, her vital signs were as follows: body temperature, 36°C; heart rate, 177 bpm; respiration rate, 70 beaths per minute; blood pressure, 106/70 mm Hg; and oxygen saturation (S_P_O_2_) at 74%.

After admission, the patient underwent endotracheal intubation and was placed on ventilatory support (SIMV/PC model: F_i_O_2_, 72%; PEEP, 13 cm H_2_O; SIMV frequency, 35 beaths/min; PC above PEEP, 24 cm H_2_O), which stabilized her S_p_O_2_ between 90% and 95%. During hospitalization, she experienced recurrent fevers and received intermittent antibiotic therapy.

**Figure 1:**
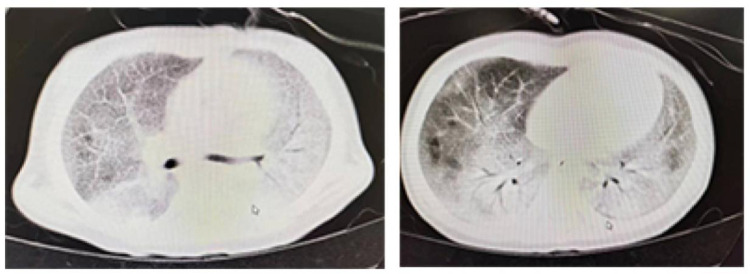
The preoperative lung CT scan of the patient showed a characteristic sign of pulmonary alveolar proteinosis, termed as “crazy paving pattern”, also known as “paving stone sign”.

Genetic testing revealed a compound heterozygous mutation in the *SLC7A7* gene. Imaging studies, including a chest x-ray, showed diffuse consolidation in both lungs, and funnel chest (pectus excavatum). Chest CT indicated a diffuse increase in lung density, with a “crazy paving” pattern (Figure 1). An echocardiogram revealed a patent ductus arteriosus with a left-to-right shunt. Arterial blood gas analysis showed a pH of 7.418, PaCO_2_ of 41.6 mm Hg, and PaO_2_ of 95.4 mm Hg, with no other abnormalities noted.

The final diagnosis included secondary pulmonary alveolar proteinosis, lysinuric protein intolerance, severe pneumonia, respiratory failure, pectus excavatum, and patent ductus arteriosus. The patient was scheduled to undergo whole-lung lavage under general anesthesia.

### Anesthetic management

For this low-birth-weight patient, our team conducted a comprehensive preoperative assessment, ultimately formulating a personalized anesthesia protocol that integrated intravenous-inhalational combined general anesthesia with tracheal intubation and an extraluminal bronchial blocker (BB) for one-lung ventilation (OLV).

**Figure 2:**
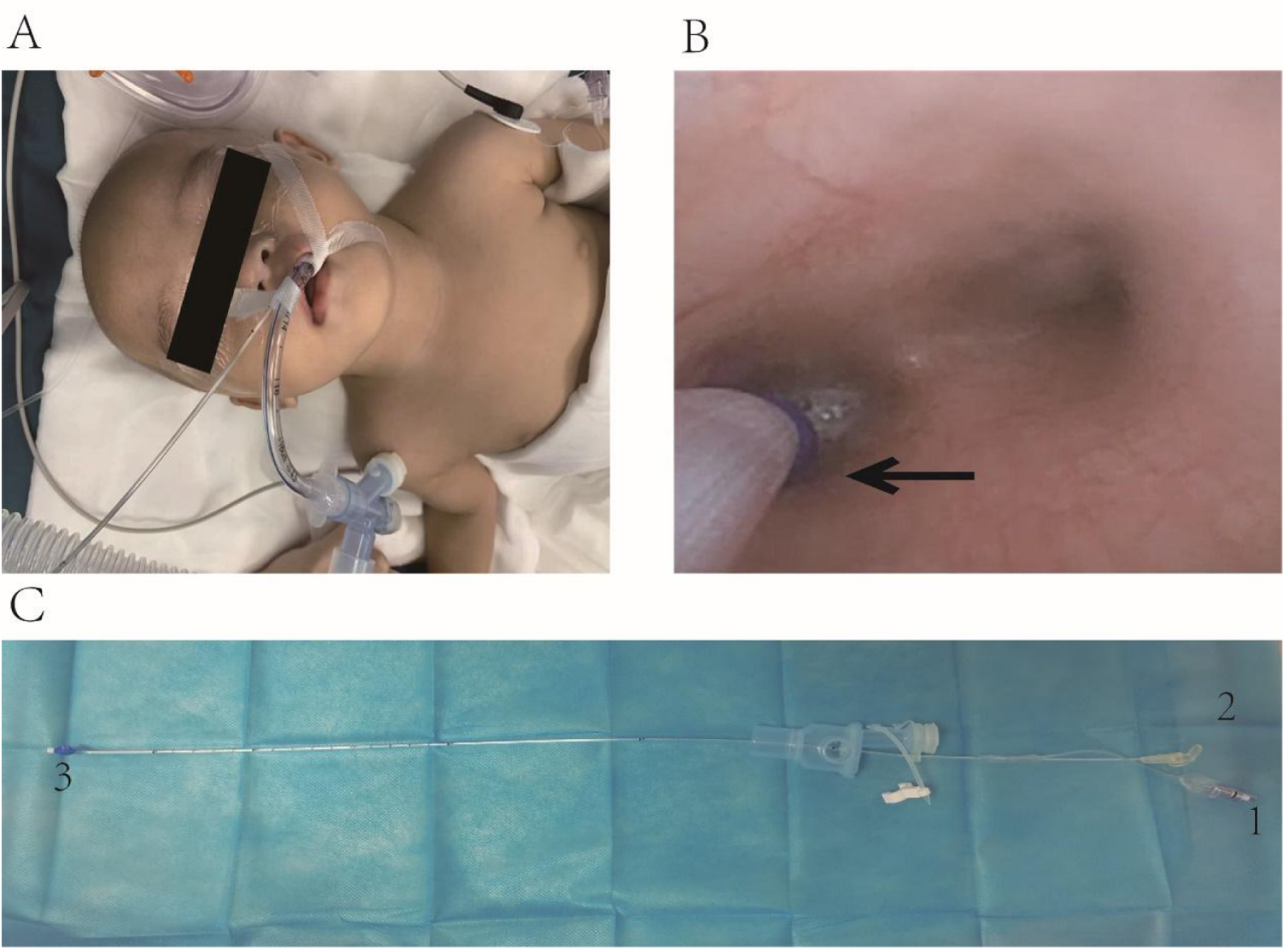
**(A)** Placing the BB outside the endotracheal tube and fixing the position. **(B)** Under direct vision using a fiberoptic bronchoscope, the balloon of the BB is positioned at the left bronchial opening, as indicated by the black arrow. **(C)** The bronchial blocker. The number 1 is the inflation port of the BB, which is connected to the balloon at the end; the number 2 is the suction port of the BB; the number 3 is the balloon and the caudal end of the BB.

Upon the patient's arrival, vital sign monitoring revealed an SpO₂ of 88%, heart rate of 136 beats/min, and blood pressure readings of 86/49 mmHg, indicative of mild hypoxia and tachycardia. Following 5 min of oxygen supplementation via the face mask, the SpO₂ gradually rebounded to 100%, establishing a favorable foundation for subsequent anesthesia induction. Subsequently, we administered midazolam 1 mg intravenously for sedation, cisatracurium besylate 1 mg for muscle relaxation, and fentanyl 20 μg for analgesia, ensuring a smooth induction process. With the aid of a videolaryngoscope, we successfully prepositioned a 5Fr ultrafine BB (internal diameter 1.7 mm, Hangzhou Tappa Medical Technology Co., Ltd., China) transglottically into the trachea, followed by rapid insertion of a 4.5-sized uncuffed endotracheal tube (internal diameter 4.5 mm, external diameter 6.5 mm) to a depth of 12 cm (Figure 2A). Confirmation of the tube's tip position was achieved by auscultation of bilateral breath sounds. Subsequently, the endotracheal tube was connected to a ventilator, initiating volume-controlled mechanical ventilation with parameters adjusted to a tidal volume of 8–10 ml/kg, respiratory rate of 26–30 beaths/min, and an I:E ratio of 1:1.5, to maintain stable ventilation. Intraoperatively, a continuous infusion of remifentanil (0.2 μg/kg/min) was administered to provide stable analgesia and sedation, complemented by a propofol infusion (5 mg/kg/h) and 1%–2% sevoflurane inhalation, constituting a multimodal anesthesia maintenance regimen.

After stabilizing the patient's oxygen saturation, we utilized a fiberoptic bronchoscope to adjust the BB cuff position through the three-way stopcock of the respiratory circuit, positioning it precisely at the left main bronchus ostium (Figure 2B). The cuff was then inflated appropriately to achieve OLV of the right lung. During this procedure, meticulous attention was paid to securing the BB and endotracheal tube to prevent displacement due to changes in patient positioning, with repeated bronchoscopic localization performed as necessary.

**Figure 3:**
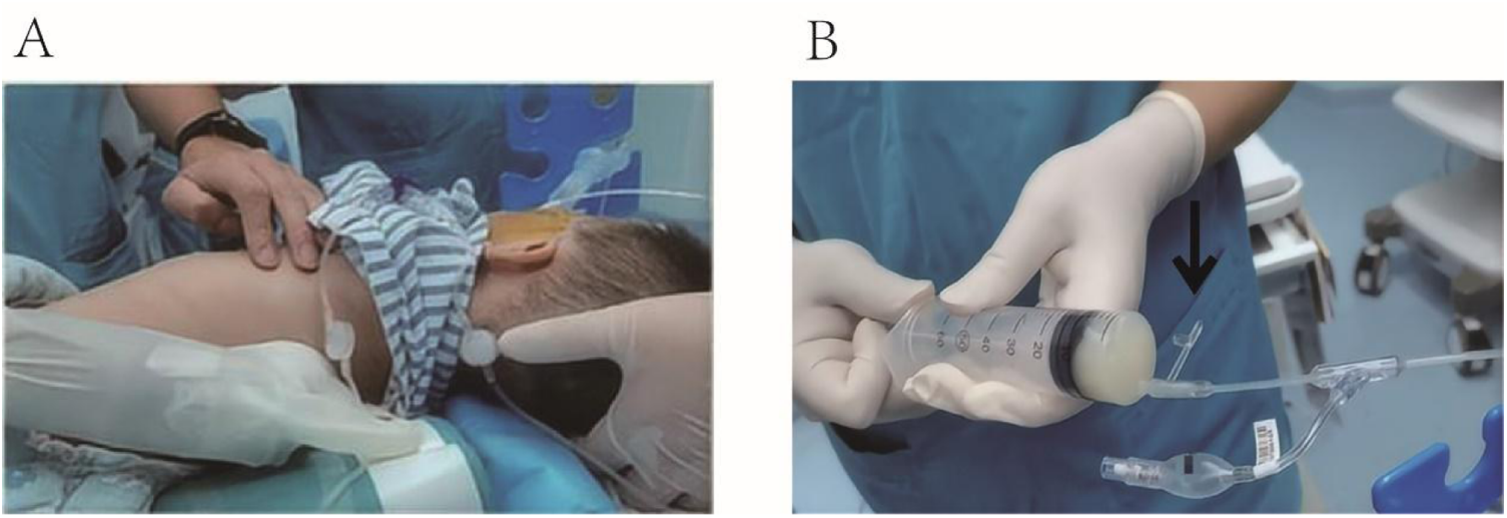
**(A)** A nurse used a high-frequency chest wall oscillation machine to vibrate the patient's left lung area. **(B)** A syringe connected to the suction port was used to aspirate the lavage fluid, as indicated by the black arrow.

The patient was initially placed in the supine position. The surgeon injected 37°C normal saline into the patient's left lung through the suction port of the BB using a syringe. A nurse then used a high-frequency chest wall oscillation machine to vibrate the patient's left lung area for 3 min. Afterward, the patient was repositioned to the right decubitus position, and the left lung area was vibrated again for 3 min. The surgeon then aspirated the lavage fluid. This process of irrigation, vibration, and repositioning was repeated multiple times (Figure 3).

**Figure 4:**
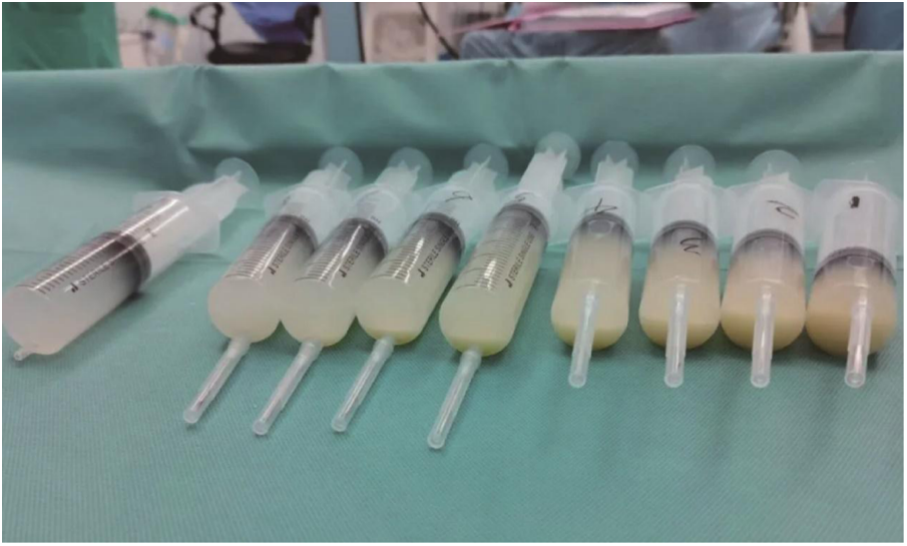
The lavage fluid, it gradually become clear from right to left.

During the procedure, the left lung was lavaged 15 times. Each lavage involved 30–60 ml of saline, totaling 730 ml of lavage fluid used. Of this, 578 ml was aspirated, leaving 152 ml retained in the lung. Lavage continued until the fluid returned clear (Figure 4). To address the retained lavage fluid and prevent potential pulmonary edema, 1.5 mg of furosemide was administered intravenously. Intraoperatively, respiratory parameters were carefully adjusted to maintain airway pressure below 30 cm H_2_O, SpO_2_ above 90%, and P_ET_CO_2_ below 60 mmHg.

The procedure lasted approximately 2 h and 18 min, and the WLL was successfully completed without any anesthetic complications. At the conclusion of the operation, the balloon of the BB was deflated, and the BB was removed from the airway. The patient was then transferred to the pediatric intensive care unit (PICU) with the endotracheal tube still in place. Postoperative assessments showed an improved oxygenation index compared to preoperative levels. Chest x-rays taken after the surgery revealed diffuse consolidation in both lungs, with slight improvement compared to the preoperative images.

## Discussion

Pulmonary alveolar proteinosis (PAP) is a rare disease with an incidence of approximately 0.2 per million inhabitants per year (8). It is characterized by the significant accumulation of lipoprotein substances within the alveoli, leading to impaired gas exchange and ventilation-perfusion imbalance, which can progress to respiratory failure and, in severe cases, death (9). Currently, whole lung lavage (WLL) is considered the most effective treatment for alleviating the symptoms of PAP, aimed at removing the accumulated lipoprotein material from the alveoli to restore gas exchange (10, 11). While electronic fiberscopic alveolar lavage performed is less invasive, it is associated with limited volume capacity and suboptimal efficacy (12). The success of this procedure, especially in infant patients, hinges on effective lung isolation to facilitate one-lung ventilation (OLV) and optimize oxygenation while the other lung undergoes lavage.

In the case we managed, the patient's young age, low body weight, and multiple comorbidities required a tailored approach to anesthesia. The approach involved a combination of intravenous and inhalation anesthesia with tracheal intubation, alongside OLV using an external tracheal catheter with a bronchial blocker (BB). This technique had not been previously described in infants for WLL. The BB we used is a small catheter with an inflatable balloon at its distal end and a suction port connected to the same end with a catheter diameter of 1.7 mm (Figure 2C). It enables lung isolation and allows lavage of lung contents through the suction port. The BB is minimally invasive and offers the advantages of easy and flexible placement, providing precise lung isolation, which makes it particularly beneficial for use in small children (13).

Previous studies have explored various lung isolation techniques in pediatric WLL, each with distinct advantages and limitations. Paquet and Karsli (14) introduced a method involving two tracheal tubes to simulate the function of a double-lumen endotracheal tube (DLT) in a 2-year-old patient. In this approach, a 3.5 mm internal diameter (ID) cuffed tube was inserted into one mainstem bronchus, while a 3.0 mm ID cuffed tube was positioned in the trachea. Although effective for lung isolation, this technique requires sufficient airway size to accommodate both tubes, which limits its use in smaller children. Other techniques, such as those described by Moazam et al. (15) and Reiter et al. (16), involve inserting a cuffed tracheal tube along with a pulmonary artery catheter (PAC) placed endobronchially. The PAC, with an external diameter of 1.67–2.3 mm, can also be used in infants but may not provide the same level of flexibility or ease of adjustment as other methods. Additionally, Wilson et al. (17) utilized a urinary catheter threaded alongside the tracheal tube, with an inflated balloon for lung isolation. While this method may be feasible in certain cases, it presents challenges in terms of catheter placement and the potential for airway trauma.

The traditional DLT is considered the gold standard for lung isolation in older children, but it has limitations in pediatric patients with small airways. The smallest available DLT, the 26F (with an inner diameter of 8.7 mm), is only suitable for children over 8 years of age. In very small children, the Marraro DLT has been used for OLV (18), but it is not ideal for facilitating lung lavage. The Marraro DLT is uncuffed, which impedes effective isolation of the lung being lavaged, and increases the risk of contamination of the ventilated lung. This is a critical consideration, as adequate lung isolation is paramount during WLL to ensure the lavage fluid does not enter the contralateral lung.

In contrast, the BB provides a significant advantage in these cases. The BB is a more flexible and minimally invasive solution that allows precise lung isolation with fewer complications related to airway trauma or inadequate ventilation. It can be easily adjusted under direct visualization using a fiberoptic bronchoscope, which enhances the likelihood of successful placement, especially in smaller pediatric patients. Moreover, the use of a BB avoids the need for more invasive techniques like extracorporeal membrane oxygenation (ECMO), which carries significant risks and may not always be available (19). The ability to achieve effective lung isolation with the BB, particularly in infants and small children, makes it a valuable alternative to more restrictive and invasive methods, offering a safer and less complex approach to managing pediatric WLL. These comparative advantages highlight the utility of the BB in pediatric WLL and suggest that it could be a preferable choice, particularly in infants and children with small airways or those requiring a more flexible approach to lung isolation.

In the case, the BB placement was stable throughout the procedure, and blood oxygenation levels were successfully maintained above 90%, indicating effective lung isolation. The use of a BB in infants is technically challenging due to the limited airway size, which can complicate both placement and the maintenance of effective ventilation. Given the small internal diameter of the tracheal catheter (internal diameter 4.5 mm) used in this child, inserting the BB into the catheter may increase airway pressure, reduce sufficient ventilation, and reduce the operational space for the bronchoscope. Compared with other lung isolation techniques, the BB's flexible placement is better suited to infant lung anatomy. Under direct visualization with a fiberoptic bronchoscope, the position can be easily adjusted, resulting in a higher success rate for effective bronchial blockade. If lavage of the contralateral lung is required, the BB can be repositioned under direct vision. This method is easier to adjust than other techniques and causes less airway trauma, while still ensuring the efficacy of the lavage.

After a thorough preoperative discussion, our team determined that the patient's left lung was severely affected, and we opted to lavage the left lung, as the lung with milder lesions would better tolerate OLV during the procedure. Following the injection of saline into the surgical lung, we employed a high-frequency chest wall oscillation machine to vibrate the chest and back on the surgical side. This oscillating action helped to fully mix the lung contents, facilitating the more effective removal of lipoprotein substances from the lung (20). For these patients, the duration of mechanical ventilation following surgery typically ranges from 2 to 6 h and patients who undergo bilateral lung lavage often require a longer observation period (21). In this case, the child required ventilator support prior to surgery due to poor lung function. As a result, endotracheal intubation was performed, and the patient was transferred to the PICU for further monitoring.

In addition, the patient's positioning during WLL plays a critical role in the success of the procedure and the patient's overall stability. Positioning the lavaged lung in the uppermost position allows gravity to aid in draining the lavage fluid, which not only facilitates the effective removal of accumulated debris and surfactant but also helps to minimize intrapulmonary shunting maintain better ventilation-perfusion matching. By reducing shunting, hypoxemia is lessened, contributing to improved oxygenation throughout the procedure (22, 23). Proper patient positioning, combined with vigilant monitoring of oxygen saturation and lung compliance, is essential to optimizing outcomes during WLL. When the patient's position is changed during the procedure, it is necessary to manage the position of the tracheal tube and BB to avoid displacement. Displacement of the bronchial blocker can lead to the lavage fluid flowing into the ventilated lung, causing severe hypoxemia. Due to the patient's alveolar diffusion dysfunction, the effectiveness of inhalation anesthesia may be diminished, making intravenous anesthesia the preferred method (24). Intravenous anesthesia ensures a more reliable delivery of anesthetic agents, bypassing the compromised gas exchange in the lungs, and provides better control over anesthesia depth and stability, reducing the risk of hypoxemia during the procedure.

While the use of the BB provides significant advantages in terms of flexibility and reduced airway trauma, there are inherent risks and limitations associated with its use. The small size of the pediatric airway, particularly in infants with low body weight, can complicate the placement of the BB and increase the risk of airway pressure, which may lead to inadequate ventilation or airway injury. Additionally, the BB requires precise placement and constant monitoring to ensure that it remains in the correct position during the procedure. The skill and experience of the anesthesiologist are crucial to minimizing these risks and ensuring a successful outcome. Further studies are needed to evaluate the long-term safety and effectiveness of this technique in a broader pediatric population.

## Conclusion

The use of a BB for one-lung ventilation in an infant undergoing WLL offers a novel and effective method for lung isolation. It minimizes airway trauma, allows flexible placement, and adapts to the small pediatric airway, making it a viable alternative to more invasive techniques like DLTs. However, successful use of the BB requires skilled placement and careful management to avoid complications, particularly in infants with limited airway size. Further research is needed to assess the long-term safety and effectiveness of this technique in a broader pediatric population, including those with varying levels of lung pathology. Standardization of the technique and its application across different medical centers could help optimize its use and offer a less invasive, more adaptable option for managing complex airway cases in young patients with pulmonary disorders.

## Data Availability

The original contributions presented in the study are included in the article/Supplementary Material, further inquiries can be directed to the corresponding author.
